# Harvard Odontological Society

**Published:** 1902-02

**Authors:** 


### HARVARD ODONTOLOGICAL SOCIETY.

THE Harvard Odontological Society held its regular meeting April 25, 1901, President Paul in the chair.

*President Paul.*—It is a good thing sometimes to have one of the younger men in the profession as our essayist. They are very apt to bring us something new, something original. I have the pleasure of introducing one of our younger members, Dr. Lawrence W. Baker, whose subject is "Facial and Dental Harmony."

(For Dr. Baker's paper, see page 69.)

### DISCUSSION.

*President Paul.*—This is a subject that certainly interests us all. Those of you who are making plates for three and five dollars a set have the opportunity to see how you can make them better and give more value for the money.

*Dr. Moriarty.*—I have been very much interested in what Dr. Baker has said, and we have got many points from the cases that he has presented here to-night. At the same time, while we all may aim for artistic effects in the mouths of our patients, in the majority of cases we cannot reach this result, because of the obstinacy of the patients themselves. While some of our patients will want teeth that are artistic, that are suitable for their age, the majority of them, women especially, will want teeth that are ten to twenty years younger looking than their teeth were at the time they were taken out.

I thoroughly believe with the essayist that more or less judicious grinding is what is required in the making up of artificial dentures to make them harmonize with the facial expression. Teeth should not be put up in the mouth as they come to us from the depots.

I am glad to have heard Dr. Baker's paper and to express my appreciation of it.

*Dr. Werner.*—I came purposely because I saw from the card that a young man was lucky enough to be drawn as an essayist. I wish it would happen oftener. Young men are elected members of this Society, and perhaps three to five years may elapse before they are drawn as essayists. During that time they are apt to move in the same limited circle without attempting to make investigations for themselves, and they dry up. The essayist's work as shown here to-night is most favorable and complimentary to himself, as it proves that he is not of that kind. He has not been standing still; he has been progressing, and just see what a few years on a little original work can do! Some of the older practitioners may say that is just what we are doing in many of our pieces, we are trying to make them as natural as possible, but, as Dr. Moriarty has said, it is just what the patient does not want done, and whatever success we make is often in spite of the patient; many times we do get their consent. Within a few years such a case was forcibly presented to me in a rather extensive case of bridge-work for an elderly lady. It was on the left upper side, extending from the central to the second molar. She was sixty years old, and positively selected teeth that in color and shape were suitable for a person eighteen to twenty-five, and I was not able to induce her to take teeth that would have been more suitable to her. She was highly pleased with the work when the case was done; nevertheless, from an artistic stand-point, it was radically wrong. You have to argue with your patients and try to make them believe that those teeth that look very badly out of the mouth will look very natural in the mouth, and that many of those handsome specimen cases are wholly unsuited for the mouth.

In the making of partial cases there are many little things that have to be considered, such as receding gums, irregularities, stainings, and other points. Dr. Baker has shown very conclusively what can be done with a little judgment in making a denture correspond to the rest of the mouth. The majority of the artificial teeth that we see are abominably abnormal,—they are straight up and down, like children's wooden soldiers; they are many times too white and glossy, are often too small, they seldom show any wear, and, in fact, it seems to be the aim to make them as different from the natural teeth as possible. What we need to do is to correct this, grind and set them up so that they will look badly on the case, and then we shall find that they look very natural in the mouth.

I wish every young man could be so fortunate as to be drawn as an essayist in the first few years that he is a member of this Society; we should then have more of this original work, which would be of great benefit to the Society as well as to the young man himself.

*Dr. Bigelow.*—I am not one of the older members, nor yet one of the younger members of the organization, but I would like to express my appreciation of Dr. Baker's paper.

I think usually the question of utility—which is perhaps a strain away from the subject of the paper—is the first one that we have to consider when we are introducing artificial teeth into the mouth, and if we study very carefully the question of utility, we are many times, without knowing it, doing just the things which the essayist recommends.

Dr. Baker has laid a great deal of stress on the grinding of the cutting edge and the festooning of the gum, but he has not said so much about the relative position of the teeth to each other and to the complete dental arch. I myself have many times destroyed the sameness or evenness across the front of the mouth by taking off a central, say, and placing it in a position to more closely occlude with its opponent, and by cutting off something from the length of the centrals, or the laterals, or the cuspids, as the case might require. In addition to such changes I take into consideration the color of the teeth in making my selection, sometimes having two centrals of different shades, and having the laterals two or three shades darker than the centrals, and the cuspids two or three shades darker than the laterals, the cuspids almost always having something of a yellowish tinge. I do not go back of the cuspids in this treatment, except so far as utility is considered. I believe, too, that a great deal of comfort can be obtained for the patient by the grinding of the molars, taking off those sharp cusps. Certainly to people in advanced life it is the one thing that is going to give them a feeling of comfort in their mastication. Destroying the glaze of the enamel is another thing which adds to the general natural effect. If these things are done carefully, you will get the greatest amount of utility from your work and at the same time get some of the artistic results which the essayist has referred to, instead of having teeth which have every appearance of being artificial.

What I have said is not to take issue with Dr. Baker, but simply to bring out the fact that the arrangement of the teeth, as well as the grinding and the selection of teeth of different shades, are things to be considered in our attempts to get these desirable effects.

*Dr. Moffatt.*—I think Dr. Baker ought to be congratulated on the manner in which he has brought out the artificial part of our work. We had a member in this Society some time ago who seemed to advocate letting this part of our work be done by others; he thought that dentists ought to do operating principally and let the mechanical part of it go to outsiders who do nothing else. I think that is a great mistake, although I think the tendency is in that direction. Many dentists do not attempt to do mechanical work. They simply take the impressions and send their work down to the dental laboratory, where it is turned over to boys of from sixteen to twenty-five who have no artistic conception or perception, and who set the teeth up according to their own inartistic ideas; then they are sent to the dentist and tried in, and then sent back to the dental laboratory again to be finished, the men who make the plate never having seen the patient; and that is the reason why we do not have more success with some of our work. I think the younger men ought to learn to do their own mechanical work, and not be afraid of putting in some time to accomplish that end. There are a good many young men whose time is not fully occupied in the first few years of their practice, and instead of sitting down and waiting for some one to come in, they should devote that time to improving their skill in mechanical and other lines. I do not think there are very many really first-class mechanical workmen among the dentists to-day, and those who are first class have little difficulty in keeping their time occupied.

In regard to prices, sarcastic reference has been made to plates advertised for from three to five dollars, but if a man can do really artistic work, he can practically get his own prices, and there is no reason why it should not be just as profitable as any other branch of our work.

I think Ash's teeth are more natural looking than S. S. White's or the Consolidated. These can be obtained in Boston, and those of you who have not used them will be surprised at the artistic appearance that they present compared with the others. It has been said that patients do not want these natural teeth, and insist on having something entirely unsuited to them. I think we make a mistake in yielding to their wishes so readily. In the majority of instances they are not talked to sufficiently to have them try an artistic set, and I think if we would only devote a little time in explaining the facts to patients, they will, after a while, consent to have what they ought to have.

In this picture of Dr. Baker's nothing is brought out more prominently than the poor quality of expression in "store teeth." They apparently possess about the same appearance all through; they are too set and even, too much like each other, and you will find it so in most of the teeth that are made in moulds. They cannot get the sharp angles that Dr. Baker has brought out here with his grinding. I was told at White's that at one time they made these artistic teeth and could not sell them. It was claimed that patients would not have them. I think it is our fault. If we know what we are about, and put it strong enough, they will take them, and after they get them they will be better satisfied than if they had the plain, white-looking, characterless teeth.

*Dr. L. W. Baker.*—This is the only case we have of an upper and under denture in occlusion, and it is ground thoroughly, as Dr. Bigelow spoke of, and I think we can get a great deal of comfort for our patients by spending a little time to get a perfect occlusion. That is a point that I got from Dr. H. A. Baker, and it is my custom to grind them on the articulator so that they will "bite hairs," so to speak, at all points. I did not go into that matter deeply in the paper, as I did not think it was quite in the special line that I had selected.

*Dr. Starratt.*—While the doctor is on his feet, will he kindly tell us how he stains those teeth to imitate discolorations?

*Dr. L. W. Baker.*—I do not know very much about staining, but you can get the effect I spoke of by using a chestnut-brown mineral paint, which you can get at any of the art stores. It is painted on and the tooth is baked for about a minute in an electric furnace. I have tried scratching the stained parts with a knife for some time, and it seems to hold. I would use stained teeth especially in partial work. You know it is impossible to get the shades of the natural teeth in our artificial work, but by just putting these little stains around the gums and on the cutting edges we get a very pleasing effect.

*Dr. Werner.*—Has the essayist done anything in taking off the gloss with hydrochloric acid?

*Dr. L. W. Baker.*—I have not in any of these cases. As I stated, I am really just starting in on this line, and that is one of the points that I intend investigating later.

*Dr. Werner.*—I think some very good effects can be obtained in that way. I have seen cases where teeth from the store have been placed in hydrochloric acid, and in from half a minute to two or three minutes that shine is all taken off, and when they are dried on the model they look very much over-doctored, but they have a very natural appearance in the mouth.

*Dr. H. A. Baker.*—I hardly came prepared to say anything, as I did not know that I was coming here until about half-past five, at which time my son asked me if I would like to come down and hear his paper.

I am rather peculiarly situated in this matter; first, if I should speak complimentary of him, it might seem as if I were praising my son. On the other hand, if I should criticise the paper, they might say I was jealous. But after all, I think those of you who know me best understand pretty well that when I am discussing a paper I am apt to say what I think of it without fear or favor.

After my son got up his specimens for "Facial and Dental Harmony" he asked my opinion of them. My reply was, "Your types are very good, but in the work there is great chance for improvement;" but as he presents them here this evening the fault seems to be rectified.

This subject which he has chosen for to-night is one that I have paid a great deal of attention to all through my practice, and the second paper I ever read before a dental society was on this line under the title of "Æsthetics in Dentistry," and one of the principal features emphasized was the fulness over the cuspids and the line which is presented on the upper maxilla, which he has shown here to-night. This I worked out by a study of the skull, and, so far as I know, was original with me and was done for the purpose of restoring the features to that of normal.

While my son was working on this subject I had occasion to make an upper and under set for quite an elderly lady. For that case I used quite dark teeth, making them a little irregular and grinding the cutting edges to imitate teeth having been considerably worn. After putting them in I called Dr. Howe and Dr. Lawrence in to see them in the patient's mouth. They looked at them carefully, but made no remarks about them in the presence of the patient; but when they got into the other room I heard Lawrence make the remark, "The old man has made a pretty natural set of teeth, hasn't he?" There have been a few points brought out to-night which I wish to speak about. One is in regard to our patients not wishing artistic work. I think it depends altogether on how you approach them on the subject. If we were to put in dark, irregular teeth, with the cutting edges ground, etc., without making any remark upon it, they would refuse to wear them. First, you must educate them by defining the difference between white, even, and small artificial teeth and the natural teeth as we find them according to the age of the patient; for instance, how an elderly person looks wearing a wig intended for a younger person. The lack of harmony is most noticeable also in people who dye their hair. I think that there is one fact that always accompanies the latter,—the person who dyes his hair never deceives any one but himself.

To me the most striking illustration the essayist has shown to-night was in a photograph of the elderly lady with white artificial teeth on one side and natural on the other, when he covered each side alternately with a card. The latter explains the whole matter. It is not a hard thing to convince intelligent patients of these errors.

Another thing I consider of vital importance, and that is the occlusion, as Dr. Bigelow has stated, "for utility." Many a time I have seen an upper and under set of teeth which had only three or four points of contact. They should articulate so that you could not see daylight between them anywhere. Another great fault is in getting the upper molar teeth too long in double sets. If length is required to fill space at this point, it should be in the lower molars. Another point which I think dentists, as a rule, pay very little attention to, is the contour of the gum. This is the principal point where we get expression, especially about the cuspid region.

I wish to compliment the essayist upon his beginning, and as he improves in his work he will eventually get types which will be a credit to himself and a benefit to the rest of us. He is certainly on the right track, and I think it would be a good thing for all of the young men to follow his example and make this line a special study, so as to be able to improve their methods for doing the work that comes to them, and, therefore, be a benefit to the profession at large.

*Dr. Moffatt.*—Dr. Starratt asked about the stains. You can get boxes of stains from the manufacturers, Ash & Sons, for something like six dollars a box, which will give you almost any color that you will want to use. It is carefully painted on and baked in any of the little furnaces on the market, and, as Dr. Baker has said, it gives a very natural appearance to the tooth.

Dr. Bigelow brought out a point in regard to having the laterals darker than the centrals and the cuspids still darker, and it is a good thing to bear in mind. In the natural human teeth the front teeth are whiter than those farther back in the mouth. The air dries the surface of the front teeth during movements of the lips in ordinary conversation, and they look whiter, the same as they do after the rubber dam has been used. The back teeth do not usually get so much brushing, and in addition to that the shadows as they appear in the mouth make the back teeth look very much darker, so that by adopting Dr. Bigelow's scheme of using darker shades as you go farther back in the mouth, you get a much more natural-looking set.

If any of you have had any experience in carving teeth, you will have seen the necessity of getting a suitable color; a color which seems right with a single specimen will not do with fourteen teeth. If they are not colored with the warmer red or brown hues, you will find as soon as you get them set in a row that they look quite ghastly. One thing that is to be considered by dentists carving their own artificial teeth (which is really not very difficult, and there is no reason why any dentist cannot learn it) is that it is especially useful in single teeth. Oftentimes we need a tooth of peculiar shape, many times we need a lateral that is considerably different from anything we can find in the dental depots, and, do the best you can, the result is not satisfactory to you; but if you carve the tooth you can match the mate on the opposite side in from ten to fifteen minutes, and get just what you want, and you can also match pitted teeth.

The principal stain used in staining teeth is the oxide of titanium, which is rather an orange-yellow shade. Another is the lemon-yellow. The oxide of chromium gives a slight greenish tinge, which is used in some cases, but if you get on the side of the orange-red, or some of the warmer colors, it will go nicely for the majority of cases. Stains are baked into the body in a much more natural manner than if painted on as porcelain paint. On smooth enamel the porcelain paint may run towards certain points and make the tooth look spotted, but if mixed in the body itself it fuses in very evenly.

I think Dr. Baker brought out a very good point in regard to fillings in artificial teeth being too highly polished. In some cases they shine like a mirror, and defeat the very object for which they were put in. I think you get better results if you pack the gold in with a plugger and roughly smooth it off, and let it go at that, as that is the appearance of the average gold filling in the mouth after having been subjected to wear.

*Dr. L. W. Baker.*—I think, as father has said, Dr. Moffatt has been hitting the nail on the head so accurately, and has covered all the points in a much better manner than I could, that there is really nothing more for me to say. In regard to the point brought up by Dr. Bigelow,—that is, breaking up a set of teeth and varying the color,—I think it is a very good idea, and I am going to try one or two with that scheme. What Dr. Moffatt said about the fillings I think is partly due to the absence of the discoloration that we usually see about large fillings that have been in for some years. I intend placing a little darker stain under there, so that the discoloration will show through the enamel, which I think will give it a more natural effect.

I meant to add that this rubber-work was done by Dr. H. L. Howe. I waxed them up carefully as I wanted them, and he carried them out in the rubber, and I think you will all admit that the rubber-work is very finely done.

